# Genome assembly of *Klebsiella michiganensis* based on metagenomic next-generation sequencing reveals its genomic characteristics in population genetics and molecular epidemiology

**DOI:** 10.3389/fmicb.2025.1546594

**Published:** 2025-04-24

**Authors:** Yong Sun, Qingqing Cai, Tianyu Li, Jingbo Chen, Yuan Fang

**Affiliations:** ^1^Department of Respiratory and Critical Care Medicine, Anqing Medical Center of Anhui Medical University, Anqing Municipal Hospital, Anqing, China; ^2^Genoxor Medical Science and Technology Inc., Shanghai, China; ^3^Faculty of Naval Medicine, Navy Medical University, Shanghai, China

**Keywords:** *Klebsiella michiganensis*, genome assembly, metagenomic next-generation sequencing, genomic characteristics, epidemiology

## Abstract

**Introduction:**

*Klebsiella michiganensis*, a significant member of the *Klebsiella oxytoca* complex, has emerged as a potential pathogen in clinical settings. Despite extensive research on the *Klebsiella pneumoniae* complex, the pathogenicity and drug resistance of the *K. oxytoca* complex remain understudied, particularly regarding the reconstruction of whole genomes from metagenomic next-generation sequencing (mNGS) data.

**Methods:**

In this study, bronchoalveolar lavage fluid (BALF) from a 55-year-old woman with a suspected right lung infection in Anhui Province, China, was analyzed using mNGS.

**Results:**

Three distinct assembly strategies were employed to reconstruct the genome of *K. michiganensis*, leading to the identification of a novel ST452 strain, KMLRT2206. Comprehensive genomic analysis of this strain and 206 clinical isolates (genomes downloaded from public databases) revealed the population structure, distribution of drug resistance genes, and virulence factors of *K. michiganensis*. The results demonstrated significant genetic diversity, with the species divided into three major clades, each exhibiting distinct patterns of drug resistance and virulence genes. Notably, 38.6% of the strains harbored the *bla*_*OXY–*1–1_ gene, highlighting a potential threat of drug resistance. While virulence gene distribution was not correlated with sequence type (ST), significant differences were observed among clades.

**Conclusion:**

This study underscores the value of mNGS combined with optimized assembly strategies for accurate species identification within the *K. oxytoca* complex, providing critical insights for clinical pathogen detection and epidemiological surveillance.

## Introduction

*Klebsiella* is a gram-negative, rod-shaped, facultative anaerobic organism. Known for its capability to produce polysaccharide capsules, it possesses a defense mechanism against the host immune responses. *Klebsiella* has been isolated and characterized from diverse environmental habitats, including soil, food, plants, insects, and water. However, it is primarily recognized for its associated with hospital-acquired infections (Dantur et al., 2018; Hu et al., 2022). *Klebsiella michiganensis* is an important member of the *Klebsiella oxytoca* complex within the *Klebsiella* genus. Strains of the *K. oxytoca* complex are non-spore-forming and non-motile, forming smooth, round, dome-shaped, and glistening colonies on agar plates (Merla et al., 2019). *K. michiganensis* was first discovered in 2012 in a toothbrush holder in a Michigan household and exhibits biochemical characteristics consistent with the *Klebsiella* genus (Saha et al., 2013). This emerging pathogen has since been reported in clinical settings worldwide (Kula et al., 2024; López-Camacho et al., 2025; Yamada et al., 2024). Drug-resistant strains of *K. michiganensis* have been isolated from the abdominal fistula, sputum, blood, and rectal swabs (Chapman et al., 2020). The species demonstrates various resistance mechanisms similar to other pathogenic *Klebsiella* species, including genome- and plasmid-mediated extended-spectrum β-lactamases, carbapenemases, and drug efflux pumps (Abed et al., 2021; Flerlage et al., 2020; Zhang et al., 2021).

Next-generation sequencing (NGS) provides valuable data for epidemiological monitoring due to its high-throughput capabilities, particularly in reconstructing entire target genome from metagenomic NGS (mNGS) data. This information is crucial for clinical practice, enabling the identification of pathogenic strains and understanding their characteristics. Advances in NGS technology have revealed mechanisms by which *Klebsiella* invades the host immune system and causes disease, including studies on lipopolysaccharide, fimbriae, outer membrane proteins, siderophores, and allantoin metabolism related to virulence and genomics in *K. pneumoniae* (Hu et al., 2022; Igo and Schaffner, 2022).

*K. michiganensis* shares the closest phylogenetic relationship with *K. oxytoca*, with 99% similarity in the 16S rRNA gene sequences of two species (Yang et al., 2022). Despite the use of advanced technologies such as matrix-assisted laser desorption/ionization time-of-flight mass spectrometry (MALDI-TOF MS) and 16S rRNA gene sequencing in clinical laboratories, *K. michiganensis* is often misidentified as *K. oxytoca*. In 2013, MALDI-TOF MS misidentified *K. michiganensis*, likely because this species, first discovered in 2012, had not yet been included in the database. With updated dataset, MALDI-TOF MS can now accurately identify the species within the *K. oxytoca* complex (Merla et al., 2019). For example, strain 12084 and strain K210011, isolated from patients’ sputum samples and rectal swabs were initially identified as *K. oxytoca* based on MALDI-TOF/MS. Subsequent whole-genome analysis confirmed these strains as *K. michiganensis* (Li et al., 2022). Despite recent publications on the genome of *K. michiganensis*, there remains a lack of research on the direct reconstruction of high-quality genomes of *K. michiganensis* from mNGS data. Therefore, our study focused on the genomic reconstruction of *K. michiganensis* from clinical metagenomic sequencing data and conducted a comprehensive genomic analysis of global clinical isolates to address critical gaps in understanding its pathogenicity, drug resistance, and population structure, while also providing valuable insights for clinical pathogen detection and epidemiological surveillance.

## Materials and methods

### Clinical specimen

**FIGURE 1:**
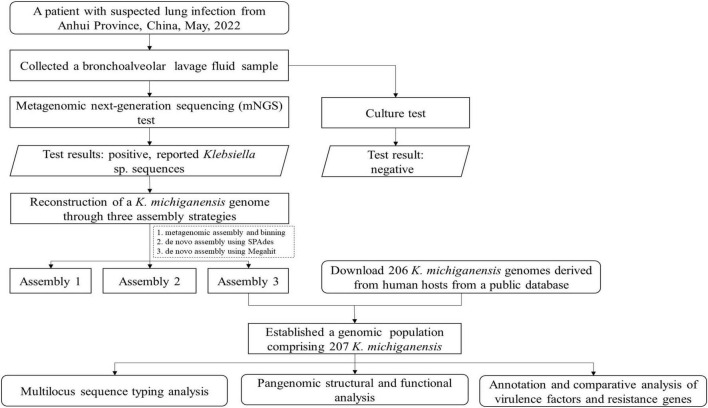
Schematic workflow of the research methodology.

Figure 1 depicts the analytical workflow of this study. A BALF sample was collected from a 55-year-old woman with a suspected right lung infection in Anqing Medical Center of Anhui Medical University, China, in May 2022. Upon admission, the patient received empirical antibiotic treatment. During the diagnostic process, bronchoscopy and pathogen culture were performed. With patient’s consent, the BALF sample was sent to Genoxor Medical & Science Technology Inc. (Shanghai, China) for mNGS test. After 48 h, the BALF cultivation test returned negative results, while the mNGS test identified numerous *Klebsiella* sp. sequences, primarily including *Klebsiella michiganensis*, *Klebsiella oxytoca*, *Klebsiella pneumoniae*, and *Klebsiella* aerogenes. Following treatment with Piperacillin Sodium and Tazobactam Sodium (4.5 g, IV, tid), the patient was discharged three days later with improved symptoms.

### Metagenomic sequencing and taxonomic profiling

DNA was extracted from the BALF sample using a TIANamp Micro DNA Kit (DP710-T2A, TIANGEN BIOTECH) according to the manufacturer’s instructions. Metagenomic libraries were then constructed using the Hieff NGS OnePot Pro DNA Library Prep Kit for Illumina (Yeasen Biotech, China). Sequencing was performed in a 75-bp single-end mode on a NextSeq 550 system (Illumina Inc., USA). To ensure high-quality sequencing data, low-quality and short reads were removed (Chen et al., 2018). A quality cutoff value of Q20 was set, and reads with base quality values below Q15 at any position were discarded. Additionally, reads shorter than 50 bp were filtered out. Human sequence data were identified and removed by mapping the data to the human reference genome (hg19) using Bowtie v2.2.6 (Langmead and Salzberg, 2012) with the parameters –threads 24 –seed 100 –no-unal. The remaining data were classified using the NCBI Microbial Genome Database. To estimate the relative abundance of each species reads in the bacterial community, the taxonomy of species abundance was calculated using Kraken v2.0.9 (Wood et al., 2019). Subsequently, metaMLST (Zolfo et al., 2017) was applied for strain-level ST typing and identification of the metagenomic data. No-template control (NTC) samples were sequenced simultaneously to monitor contamination during the experiments.

### Genome assembly and characterization of the targeted pathogen

Three strategies (designated as Assembly 1–3) were employed for the genomic reconstruction of *K. michiganensis* from metagenomic reads. For Assembly 1, MetaSpades v3.15.4 (Nurk et al., 2017) was used for assembly, followed by genome binning using the binning and bin refinement modules in metaWRAP v1.3.2 (Uritskiy et al., 2018). Contig binning was performed using MetaBAT and Maxbin2, and the resulting bins were optimized and combined to generate a draft genome for a single strain. The quality of g draft genome (Assembly 1) was assessed using CheckM (Parks et al., 2015), which evaluated completeness and contamination. Taxonomic classification was performed using GTDB-tk (Chaumeil et al., 2022), confirming the draft genome as *K. michiganensis*. For Assembly 2 and Assembly 3, RefSeq genomes of 384 *K. michiganensis* isolates (NCBI TaxID: 1134687) were retrieved from the NCBI Assembly database. Reads affiliated with *K. michiganensis* were captured using BBmap and assembled using SPAdes (parameters: -t 24 -m 128 –cov-cutoff auto –isolate) (Gurevich et al., 2013) and Megahit software (Li et al., 2015), respectively. These three assembly strategies were selected because Assembly 1 is optimized for complex metagenomic data processing, Assembly 2 is tailored for specific analysis of the target strain, and Assembly 3 can efficiently handles large-scale data, enabling a more comprehensive reconstruction of *K. michiganensis* genome.

The quality of genome assemblies was evaluated using QUAST (Nurk et al., 2017) with the reference genome of the THO-011 Strain (GenBank accession: ASM1513957v1), while contamination and completeness were evaluated using CheckM (Parks et al., 2015). Then, the Pyani v0.2.11 (Leighton Pritchard et al., 2016) software was employed to accurately evaluate the Average Nucleotide Identity (ANI) among the optimal assembly genomes, its closest relatives, and the type strains of the species. For each species, two or three complete genomes retrieved from NCBI Assembly database are used for the comparative analysis. An ANI score of >95% was adopted as the criterion for species delineation (Jain et al., 2018), confirming the genome as *K. michiganensis*.

### Genome analysis of clinically related genotypes

Multilocus sequence typing (MLST) was performed using FastMLST v0.0.15 (Guerrero-Araya et al., 2021) and pubMLST database^1^ to assign the alleles number and STs. The analysis was conducted on the optimal assembled genome and 206 additional *K. michiganensis* genomes with an assembly level of scaffold or higher downloaded from the NCBI Assembly database (updated on April, 2023). These genomes were sourced from sapiens hosts and included seven conserved alleles of *K. michiganensis*, gapA, infB, mdh, pgi, phoE, rpoB, and tonB. Kleborate (Lam et al., 2021) software was used to invoke the software Kaptive (Lam et al., 2022) for K and O antigen typing. Virulence factors (VFs) genes were detected by performing a BLASTp against the VFDB database (Chen et al., 2005), with putative virulence genes screened using an e-value threshold of 1e^–20^. Additionally, resistance genes were annotated using the Resistance Gene Identifier (RGI) (Alcock et al., 2020) software, referencing the Comprehensive Antibiotic Resistance Database (CARD).

### Phylogenetic analysis and gene annotation

For the pan-genome analysis, all *K. michiganensis* genomes were uniformly annotated using Prokka v1.14.6 (Seemann, 2014). Roary v3.13.0 (Page et al., 2015) was used to detect and cluster orthologous genes (OGs) with the following parameters: -p 36 -i 90 -e -n -t 11 -s -cd 100 -a -v -z. The core gene supergene was developed through the following process. After obtaining the OG clusters, we selected the representative genes from each OG. These representative genes were then concatenated to form the core gene supergene. This supergene represents the conserved genetic information shared by all *K. michiganensis* strains and can be used for high–resolution phylogenetic analysis.

A phylogenetic tree for 207 *K michiganensis* strains was constructed using FastTree V2.1.10 and visualized using Evolview-v3 (Subramanian et al., 2019). To further understand the functions of the genes in the OGs, we extracted the amino acid sequences of the representative genes from each OG. These sequences were then annotated based on the best hits and an *e*-value threshold of 1e^–20^, by Blastp v2.9.0 + against the COG database.

## Results

### Genomic analysis of *K. michiganensis* KMLRT2206 strain

After quality control and removal of host sequences from the mNGS raw data, a total of 6,128,944 clean reads were obtained. Among these, 4,476,090 reads were aligned to all microbial sequences by Kraken. Within these aligned reads, 2,786,596 were assigned to the genus *Klebsiella*. However, 96.5% of these sequences could not be further classified to a specific species. MetaMLST detected *K. oxytoca* in the metagenomic data and reported a novel ST, namely ST 100001, with a confidence of 100%. Supplementary Table 1 is the New *K. oxytoca* ST table, which includes all the known STs as well as the new ST detected in the sample.

To identify the pathogenicity of *Klebsiella* sp., three assembly strategies were employed to reconstruct its genome. Table 1 summarizes the quality metrics for the genome assemblies. Based on total contig size and N50, the optimal genome assembly was generated by Assembly 3, which produced 125 contigs longer than 500 bp, with a total length of approximately 5.86 Mb and an N50 of 118,958 bp. Assembly 3 outperformed Assembly 1 and 2 in terms of N50 and Largest contig metrics. With a GC content of 56.06%, Assembly 3 was selected for subsequent phylogenomic and pan-genomic analyses, and the strain was designated KMLRT2206. The KMLRT2206 genome contains 5,422 protein-coding sequences and 15 rRNA genes.

**TABLE 1:** Comparison of genome assemblies of an *K. michiganensis* strain recovered from the metagenome data.

	Assembly 1	Assembly 2	Assembly 3
No. of contigs > 500 bp	233	305	125
No. of contigs > 10,000 bp	150	156	71
N50 (bp)	38,615	34,389	118,958
NGA50 (bp)	281,23	28,123	70,564
Largest contig (bp)	186,901	186,901	424,791
Total length (bp)	5,700,083	5,850,327	5,860,284
Average coverage depth	117.19	118.99	118.40
GC (%)	56.11	56.04	56.06
Genome fraction (%)	88.74	90.048	90.232
Completeness (%)	98.81	100	99.70
Contamination (%)	0.22	0.22	0.22
No. of CDSs	5,283	5,346	5,422
No. of rRNAs	11	17	15

No., number; bp, base pair; N50, the length of the shortest contig when the cumulative length reaches 50% of the total genome length; NGA50, calculated based on the genome-adjusted; CDSs, coding DNA sequences; rRNAs, ribosomal RNAs.

**FIGURE 2:**
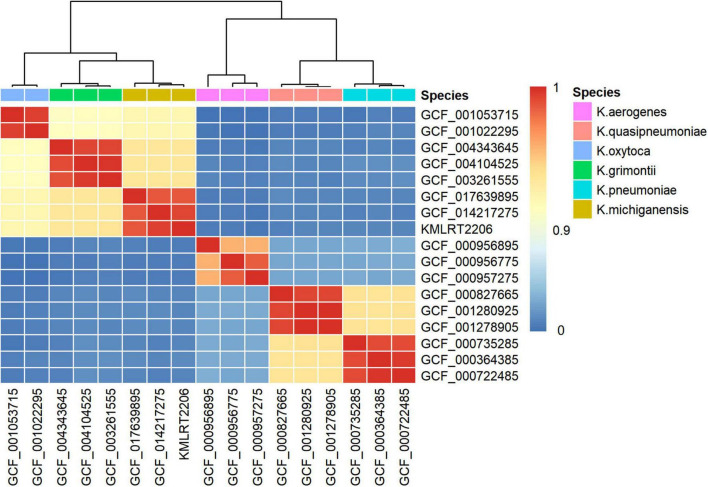
Species identification of the *K. michiganensis* strain KMLRT2206 genome recovered from clinical metagenomics data. The heatmap displays color-coded average nucleotide identity (ANI) values between pairwise comparisons of genomes from six *Klebsiella* species. The color bar (on the right) indicates the degree of similarity between species, with values ranging from 0 to 1. Red represents high similarity, while blue denotes low similarity.

Genome-wide nucleotide sequence identity analysis was performed to infer the organismal origin of the metagenome strain. As shown in Figure 2, KMLRT2206 shared 98.79%–99.34% ANI with of *K. michiganensis* genomes, while displaying low ANI values (83.19%–93.19%) with other *Klebsiella* species (Supplementary Table 2). Since the 95% ANI threshold for species delineation, strain KMLRT2206 was reclassified as *K. michiganensis*.

### Phylogeny of the KMLRT2206 genome

MLST analysis revealed that strain KMLRT2206 possesses a previously unreported allelic profile of housekeeping genes [*gapA* (3), *infB* (8), *mdh* (16), *pgi* (21), *phoE* (107), *rpoB* (19), and *tonB* (∼18)]. Upon submission of these sequences to the PubMLST database, the strain was assigned to a novel ST, ST452. Then, MLST analysis of the remaining 206 strains classified 165 strains, with the top 10 STs being ST27 (13 strains), ST50 (10), ST85 (10), ST43 (9), ST11 (8), ST213 (8), ST29 (6), ST231 (5), ST138 (5), ST35 (4), ST84 (4), and ST205 (4). Thirty-six strains represented novel ST types, whereas six strains could not be classified due to the missing housekeeping gene fragments (Supplementary Table 3).

**FIGURE 3:**
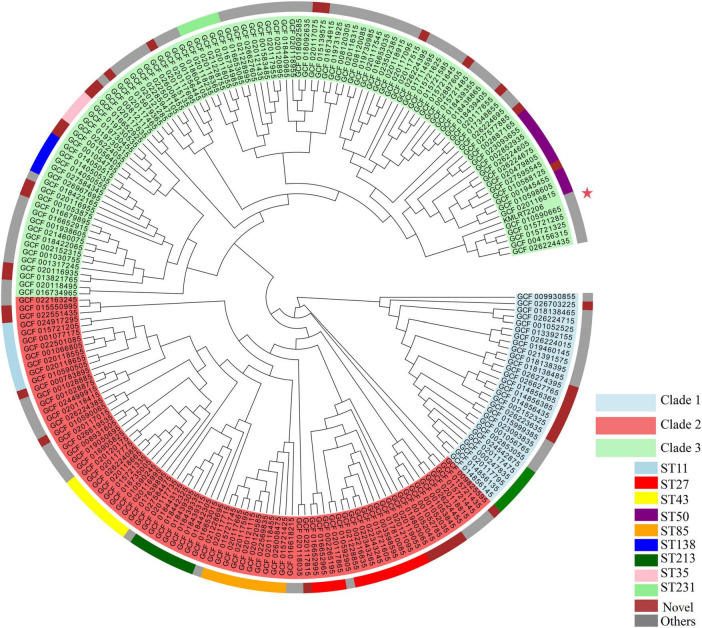
Maximum-likelihood phylogenetic tree of *K. michiganensis*. The outer circle indicates the STs of the isolates, while the inner circle represents the different clades of *K. michiganensis*. The strain KMLRT2206 is marked with a red five-pointed star.

A maximum-likelihood phylogenetic tree was constructed using the 206 *K. michiganensis* strains and KMLRT2206 (Figure 3). The tree revealed three major clades: Clade 1, Clade 2, and Clade 3. Clade 1 comprised 28 strains, including ST29 (6 strains) and 8 novel ST strains. Clade 2 contained 79 strains, with ST27, ST43, ST85, ST11, and ST213 exclusively distributed within this clade. Clade 3 included 100 strains (48.3% of the total), with ST50, ST32, and ST321 uniquely present in Clade 3. Additionally, the strain KMLRT2206 was located in Clade 3, exhibiting the closest genetic resemblance to ST32 strains (GCF010590665, GCF015721285, and GCF015721325) isolated from the United States. These findings indicate significant genetic diversity and non-clonal population structure among *K. michiganensis* strains.

Further analysis of the relationship between 207 *K. michiganensis* strains and their isolation locations and times revealed no clustering based on these factors (Supplementary Figure 1), suggesting that the evolutionary process of *K. michiganensis* is not influenced by geography or time. In terms of geographical distribution, the top six countries of isolation were the United States (53 strains), Switzerland (61), China (29), Germany (22), Australia (15) and the United Kingdom (5).

### Pan-genomic composition of *K. michiganensis*

**FIGURE 4:**
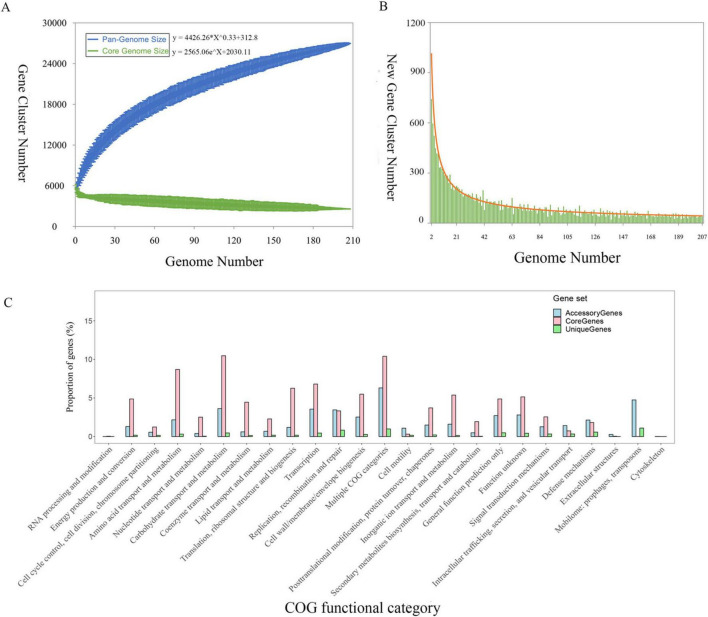
The pan-genomic structure and functional analysis of *K. michiganensis*. **(A)** Sizes of the pan-genome and core-genome. **(B)** Number of strain-specific genes per genome. **(C)** Distribution of the core genes, accessory genes, and strain-specific genes across individual COG functional categories.

Pan-genome analysis was performed using 26,966 protein-coding sequences (CDSs) across the 207 *K. michiganensis* genomes. The analysis identified 2,587 (9.6%) core OGs, 16,155 (60%) accessory genes, and 8,224 (30%) strain-specific genes, which may contribute to unique phenotypes of individual strains. The pan-genomic asymptotic curve did not reach a platform (Figure 4A), and the exponential value of the mathematical function derived from the curve exceeded 0.5 (Figure 4B), indicating that *K. michiganensis* has an open pan-genome. This suggests a high rate of gene exchange within the *K. michiganensis* species and a strong capacity to acquire novel genes from the environment or other species. The distribution of core, accessory, and unique gene sets across COG functional categories is shown in Figure 4C. Among the accessory genes, 766 genes were identified as mobile genetic elements, which play a crucial role in the transmission of virulence factors such as exotoxins and extracellular enzymes.

### Patterns of drug-resistance genes in *K. michiganensis*

A total of 37 drug-resistance genes were identified in KMLRT2206, including genes conferring resistance to aminocoumarin antibiotic [mdtC, mdtB, APH (3′)-Ia, acrD, APH (3″)-Ib, APH (6)-Id], fluoroquinolone (emrR, emrB, QnrS1), diaminopyrimidine (dfrA14), glycopeptide (vanG), nitroimidazole antibiotic (msbA), peptide antibiotic (ArnT, PmrF, eptB, bacA), tetracycline antibiotic [tet(A)] and multiple-resistant genes (baeR, LptD, acrB, marR, rsmA, oqxA, oqxB, marA, KpnE, KpnF, KpnG, CRP, OmpA, bla_*OXY–*1–1_). Analysis of 207 *K. michiganensis* strains identified 194 drug-resistance genes (Supplementary Table 4). Common resistance genes included those for aminocoumarin antibiotic (mdtB, mdtC), aminoglycoside antibiotic (acrD), peptide antibiotic (eptB), nitroimidazole antibiotic (msbA), and multiple resistance genes (PBP3 conferring resistance to β-lactam antibiotics, acrB, rsmA, KpnF, KpnG). Among the 60 β-lactamase resistance genes identified, bla_*OXY–*1–1_ gene was present in 38.6% (80/207) of strains. In addition, a variety of carbapenemase genes were detected in 60 isolates, including 14 bla_*NDM*_ genes, 12 bla_*VIM*_ genes, 5 bla_*IMP*_ genes, and 46 bla_*KPC*_ genes (Supplementary Table 4). Among them, bla_*KPC–*2_ was the most prevalent variant, accounting for 31 isolates. The co-occurrence of different carbapenemase genes was detected in two clinical isolates. We further investigated the correlation between resistance genes and evolutionary clades. The results indicated no obvious difference in the number of drug-resistance genes among different evolutionary clades (Supplementary Figure 2). However, bla_*OXY–*5–1_ was present in all 27 strains of Clade 1, while bla_*OXY–*1–1_ and bla_*OXY–*1–2_ were absent in this clade. bla_*OXY–*1–4_ was exclusively found in some strains of Clade 3 (Supplementary Figure 2).

### Patterns of virulence genes in *K. michiganensis*

Fifteen categories of virulence genes were identified in the KMLRT2206 genome, including Nutritional/Metabolic factors (90 genes), immune regulation (75), inheritance (48), effect delivery system (46), biofilm (27), regulation (18), exoxin (15), stress survival (15), motility (10), and others. Key genes in the nutritional/metabolic factors category included those related to iron carriers, such as *Yersinia* (*ybtX*, *ybtU*, *ybtT*, *ybtS*, *ybtQ*, *ybtP*, *ybtE*, *ybtA*, *irp1*) and *Enterobacterium* (*entS*, *entF*, *entE*, *entD*, *entC*, *entB*, *entA*, *fes*, *fepG*, *fepD*, *fepC*, *fepB*, *fepA*) gene clusters (Supplementary Table 5). In the immune regulation category, 26 genes were involved in lipopolysaccharides (LPS) synthesis, such as *rfbA*, *rfbB*, *rfbD*, *waaC*, and *waaF* (Supplementary Table 5). LPS, a toxin of extracorporeal alveolar macrophages, can significantly inhibit phagocytosis, alter the host immune system, lead to pathogenic and physiological changes, and interfere with other antigens. Additionally, 21 genes related to capsular polysaccharide (CPS) were identified, which help the strain evade phagocytosis and enhance its survival. CPS and LPS are key pathogenicity factors and potential targets for novel control strategies.

Annotation of 207 genomes through the VFDB database identified 510 virulence genes, with 211 common to all strains. No correlation was observed between virulence genes and ST (Supplementary Figure 3). Notably, 27 strains in Clade 1 lacked *Yersiniabactin* virulence genes, 26 strains in Clade 1 lacked *cytochrome C* virulence genes, and 23 strains in Clade 3 lacked *Yersiniabactin* virulence genes. All strains in Clade 2 possessed both *Yersiniabactin* and *cytochrome C* virulence genes (Supplementary Figure 3).

Among the 207 strains, 161 had accurately identified O antigens, including O1 (127 strains), O2a (33), and O2ac (1). The remaining 46 strains could not be classified. KMLRT2206 was identified as O1. For K antigens, 32 strains were classified into six types: K70 (11), K43 (11), K26 (4), K74 (3), and K41 (1). The remaining 175 strains, including KMLRT2206, could not be classified, with the best match for KMLRT2206 being KL107 (75.85% identity). Further analysis indicated that all ST213 and ST32 strains were identified as O2a, and the K43 was closely associated with ST43 (Supplementary Figure 3).

## Discussion

In this study, we report the genomic characteristics of *K. michiganensis* recovered from patient with pulmonary infection using mNGS technology in a hospital in Anhui Province, China, and place it within a broader range of clinical samples from 20 countries. At these two geographical scales, the overall *K. michiganensis* population exhibited rich genetic diversity, with the emergence of a large number of new STs. ST27, ST50, and ST85 are widely distributed, and strains of different STs showed differences in resistance genes, virulence genes, and antigen types.

Traditional tests based on phenotypes, biochemistry, and PCR for differentiating members of the *K. oxytoca* complex have certain limitations in sensitivity, accuracy, and speed. Culture method is the gold standard for bacterial identification, but the probability of false–negative results increases after empirical drug use (Li et al., 2021). This is consistent with the results observed in this study. 16S rRNA gene sequencing is another commonly used bacterial identification technique. Due to its low taxonomic resolution and the close genetic distance among members of the *K. oxytoca* complex, it is difficult to accurately identify species (Pan et al., 2023). Whole-genome sequencing and analysis provide the highest level of resolution for accurate bacterial species identification (Konstantinidis and Tiedje, 2005), but it is time-consuming, culture-dependent, and cannot quickly obtain all potential pathogens in clinical samples. In contrast, in this study, mNGS combined with an optimal assembly strategy was used to simultaneously conduct rapid and extensive screening of pathogens, identification of species subgroups, and determination of resistance genes without relying on culture. This not only helps clinicians to identify pathogens earlier, reduce unnecessary use of broad-spectrum antibiotics, and lower the risk of drug resistance, but also provides important reference for deciphering *K. michiganensis*.

Accurate identification of bacterial species is not only crucial for improving patient care, as it may affect the interpretation of drug susceptibility tests and thus impact treatment outcomes, but also for enhancing our understanding of bacterial epidemiology (Saxenborn et al., 2021; Voellmy et al., 2022; Wu et al., 2021). Previous study has demonstrated that the use of a core-genome MLST scheme can provide fine-scale resolution for strain discrimination of *K. pneumoniae* (Raj et al., 2022). Ikhimiukor et al. (2023) analyzed a global *K. oxytoca* dataset spanning 15 years and 18 countries, and the results showed that ST2, ST176, and ST199 were prevalent and widely spread in clinical infections. We performed MLST analysis on 207 *K. michiganensis* strains obtained from human hosts. A large number of new STs (36 strains) and unclassifiable strains (6 strains) were discovered, which fully indicates the rich genetic diversity of *K. michiganensis* (Figure 3). The phylogenetic tree divided *K. michiganensis* into three major clades, and this distribution may be related to the origin and divergence history of each clade. ST27 was the most common ST (13/207, 6.3%), followed by ST50 and ST80 (Figure 3). This result differs from that of Li et al. (2022) in their analysis of 275 *K. michiganensis* strains from human, animal, and environmental sources, where they reported that ST29 (12/275) was the most common ST. Additionally, ST29, ST43 and ST92 have been reported as predominant STs of carbapenem-resistant *K. michiganensis* (Li H. et al., 2024; Wan et al., 2023). These differences may be due to variations in sample sources, geographical regions, and sample collection times. The high prevalence of ST27 indicates its relatively frequent occurrence in the local clinical setting compared to other STs. In addition, the ST27 *K. michiganensis* strains had the highest number of resistance genes (Li et al., 2022). Consequently, enhanced surveillance of this strain is warranted to mitigate potential public health risks. There was no obvious clustering relationship between the distribution of *K. michiganensis* strains and the isolation location and time (Supplementary Figure 1). This conclusion is different from the traditional view that microbial evolution is greatly influenced by geographical and temporal factors, which may imply that this species has a special transmission mechanism or evolutionary driving force.

A correlation between β-lactamase genes and species has previously been reported, where *K. pneumoniae* is associated with the *bla*_SHV_ gene, *K. quasipneumoniae* with the *bla*_OKP_ gene, and *K. variicola* with the *bla*_LEN_ gene (Haeggman et al., 2004). In the analysis of resistance genes in this study, the *bla*_OXY–1–1_ gene was detected in 38.6% (80/207) of *K. michiganensis* strains, and it coexisted with other β-lactamase resistance genes (Supplementary Table 4), which poses a potential threat to public health. Recent study has demonstrated that *bla*_OXY–5_-carrying *K. michiganensis* exhibits higher antibiotic resistance rates, representing a highly resistant subpopulation (Li Y. et al., 2024). In our study, the clade-specific distribution pattern of *bla*_OXY–5–1_ gene was observed (100% prevalence in Clade 1) (Supplementary Table 4), suggesting a potential evolutionary link between phylogenetic divergence and antimicrobial resistance in *K. michiganensis* that warrants further investigation. It is known that sequence variations of the chromosomally encoded β-lactamase gene *bla*_OXY_ can assign the *K. oxytoca* complex to phylogroups (Brisse et al., 2016). Nine phylogroups, Ko1 to Ko9, were assigned to reflect the *bla*_OXY_ variants they carried (*bla*_OXY–1_ to *bla*_OXY–9_) (Yang et al., 2022). Previous study has used the *bla*_OXY_ variants of *bla*_OXY–1_ and *bla*_OXY–5_ to help distinguish between phylogroup Ko1 and sub-phylogroup Ko5 (Fevre et al., 2005), and the results of our article are consistent with this (Supplementary Figure 2). Cosic et al. (2021) proposed that combining *bla*_OXY_ genotyping with auxiliary gene markers enables species discrimination within the environmental *K. oxytoca* species complex. This suggests that variations in β-lactamase genes may reveal lineage divergence more rapidly than variations in housekeeping genes. In the *Klebsiella* genus, the predominant carbapenem resistance gene is class A serine β-lactamase *bla*_KPC–2_ (Chatzidimitriou et al., 2024), which is consistent with our findings (Supplementary Table 4). Notably, this distribution pattern differs from Spanish epidemiological reports documenting *bla*_VIM–1_ and *bla*_OXA–48_ as the predominant carbapenemases in *K. oxytoca* populations (Pérez-Vazquez et al., 2019). Findings revealed that *K. michiganensis* strains can co-harbor multiple carbapenemase genes (Founou et al., 2018; Sun et al., 2024; Zhang et al., 2022), demonstrating this species’ potential for accumulating antimicrobial resistance determinants. Although only a limited number of isolates carrying multiple carbapenem resistance genes were detected in our study (Supplementary Table 4).

In the analysis of antigen types, we found that the CPS K antigens of most *K. michiganensis* isolates in the population were unknown (Supplementary Table 5), which is consistent with a study from Australia (Stewart et al., 2022). Additionally, the CPS K antigen locus exhibited low prevalence (Supplementary Table 5), consistent with findings from a previous study (Biedrzycka et al., 2023). This indicates that the K antigens of most *K. michiganensis* strains remain uncharacterized, which may impede clinical serological analysis. Both ST213 and ST32 strains were identified as serotype O2a, and K43 was closely associated with ST43 (Supplementary Figure 3), suggesting that there is a certain correlation between the antigen type and the ST of the strains. Li Y. et al. (2024) have revealed the distinct distribution patterns of K and O loci between *K. michiganensis* and *K. oxytoca*. This correlation may contribute to more accurate classification and tracing of *K. michiganensis*. In the analysis of virulence genes, there was no obvious correlation between virulence genes and ST (Supplementary Figure 3), which means that the composition of virulence genes of strains cannot be inferred solely based on ST. However, the distribution of virulence genes differed among different clades of *K. michiganensis*. The *Yersiniabactin* virulence genes were not detected in any of the strains within Clade 1, but were found in Clade 2 and Clade 3 (Supplementary Figure 3). It is known that *Yersiniabactin* virulence genes can block the production of reactive oxygen species in the respiratory system, indirectly reducing the bactericidal ability of host innate immune cells and increasing the virulence of clinical infections (Paauw et al., 2009). This implies that different clades may exhibit differences in pathogenic characteristics, which may be attributed to different selective pressures experienced during the evolutionary process, leading to the acquisition or loss of virulence genes.

Currently, there are still many research gaps in our understanding of the *K. oxytoca* complex. The proportion of *K. michiganensis* strains that are insensitive to carbapenems and cephalosporins has been increasing year by year. Without careful monitoring, it is likely to pose greater challenges to treatment and infection control in the future. This study pioneered the proposal of the value of mNGS combined with the optimal assembly strategy in the accurate identification of species within the *K. oxytoca* complex. However, the reliability and broad applicability of mNGS in practical clinical applications remain to be further verified. Recent studies have shown that MALDI-TOF also has the potential to identify subgroups within bacterial species for epidemiological assessment (Cuénod et al., 2021; Giraud-Gatineau et al., 2021). Therefore, performance comparison with MALDI-TOF technology is necessary in the future. At the sample level, this study only reconstructed one strain of *K. michiganensis* from a clinical specimen collected at a hospital in Anhui Province, China, while the other isolates were derived from global clinical samples in public database. There is still room for improvement in geographical coverage and sample diversity, and it may not comprehensively reflect the genetic characteristics and distribution patterns of *K. michiganensis* under different environmental and host backgrounds. Secondly, in terms of clinical significance, since the colonization incidence, pathogenicity, as well as clinical manifestations, severity, and prognosis of species within the *K. oxytoca* complex in patients are still largely unknown, it remains to be elucidated whether the accurate species identification of *K. michiganensis* or the differentiation of the *K. oxytoca* complex has an impact on patient treatment, prognosis prediction, epidemiological monitoring, and infection control. We believe that currently, the accurate identification of species within the *K. oxytoca* complex is necessary for scientific research. This helps to improve epidemiological monitoring, precisely monitor the situation of drug resistance, and thus formulate targeted prevention and control strategies. However, it may not be necessary for routine clinical practice.

## Conclusion

In this study, mNGS was performed on a BALF sample obtained from a patient, enabling rapid and accurate pathogen identification and a comprehensive analysis of the strain’s genomic characteristics. This provided reliable information for clinicians and epidemiologists. Additionally, we conducted an in-depth analysis of population genetics and molecular epidemiological features using 206 published *K. michiganensis* genomes from human hosts, establishing a critical framework for understanding this species. Among the 207 *K. michiganensis* strains analyzed, the predominant O- antigen was O1, and the most common ST was 27. The strain KMLRT2206, identified as a novel ST452 strain, carries multiple virulence and drug-resistance genes. It belongs to Clade 3 and encodes genes for the carbapenemase and the yersiniabactin, indicating its potential pathogenicity and warranting further investigation. A limitation of this study is the potential sampling bias in genome sequencing. Future research should focus on systematically exploring the prevalence, pathogenesis, antibiotic resistance, and transmission dynamics of *K. michiganensis*.

## Data Availability

The names of the repository/repositories and accession number(s) can be found below: https://www.ncbi.nlm.nih.gov/, PRJNA1021007.

## Footnotes

1
https://pubmlst.org

## Supplementary material

Supplementary Figure 1 Maximum-likelihood phylogenetic tree of *K. michiganensis* with country and collective date information.

Supplementary Figure 2 Maximum-likelihood phylogenetic tree of *K. michiganensis* with a heatmap of antibiotic resistance gene.

Supplementary Figure 3 Maximum-likelihood phylogenetic tree of *K. michiganensis* with a heatmap of virulence gene.

Supplementary Table 1 Results of MetaML ST of BALF metagenomes.

Supplementary Table 2 Pairwise average nucleotide identity (ANI) values between genomes of distinct *Klebsiella* species.

Supplementary Table 3 Metadata of epidemiological and genomic features of all *K. michiganensis* strains used in this study.

Supplementary Table 4 Antibiotic resistance encoding genes in *K. michiganensis*.

Supplementary Table 5 Genes encoding virulence factors in *K. michiganensis*.
