# Supplementary figures and images for: Genome assembly of Klebsiella michiganensis based on metagenomic next-generation sequencing reveals its genomic characteristics in population genetics and molecular epidemiology
Source: Front Microbiol. 2025 Apr 24;16:1546594. doi: 10.3389/fmicb.2025.1546594 (PMC12058774; doi:10.3389/fmicb.2025.1546594)

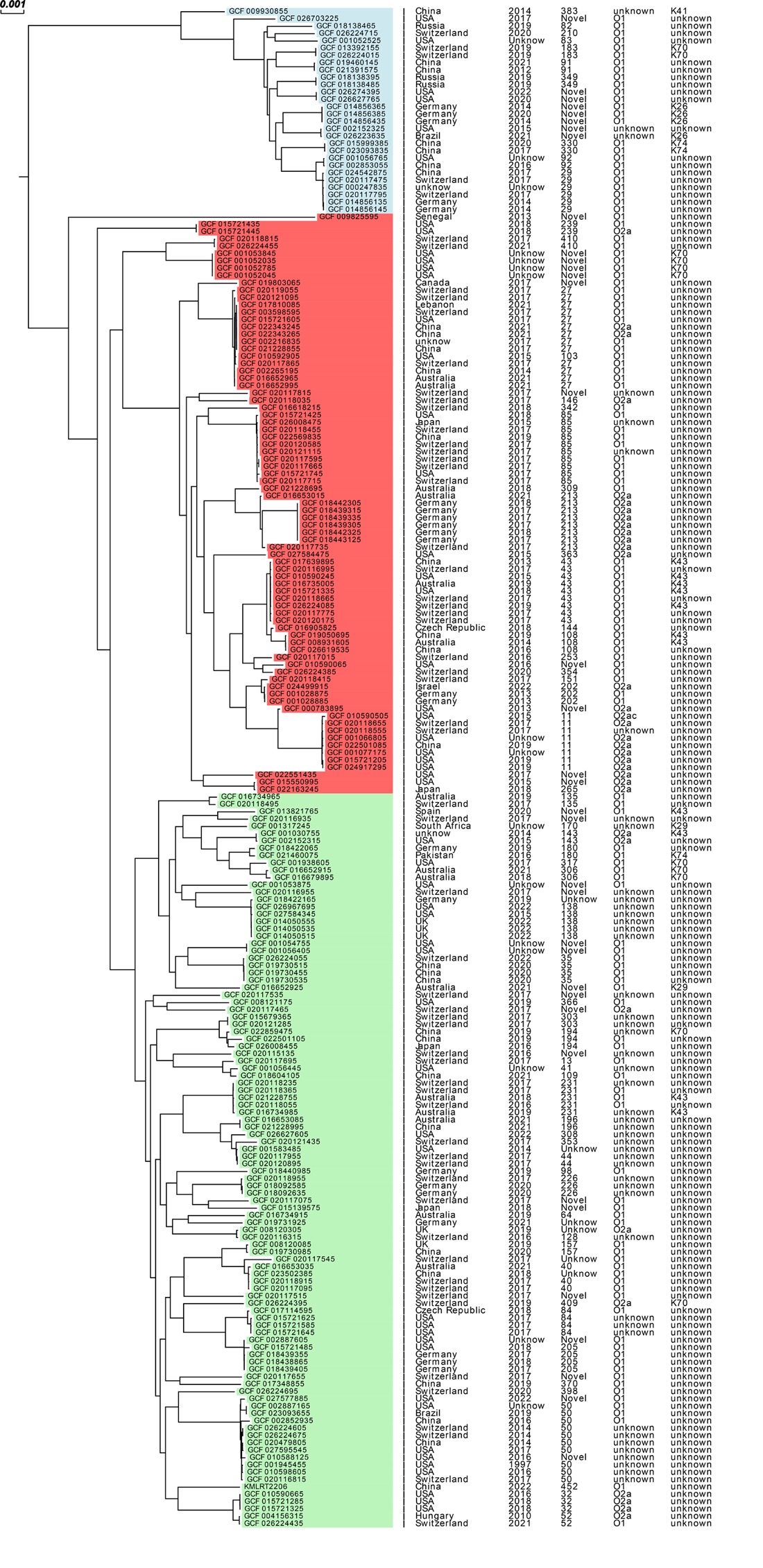

Supplement: Supplementary Figure 1 — Maximum-likelihood phylogenetic tree of K. michiganensis with country and collective date information. [file Image_1.tif]

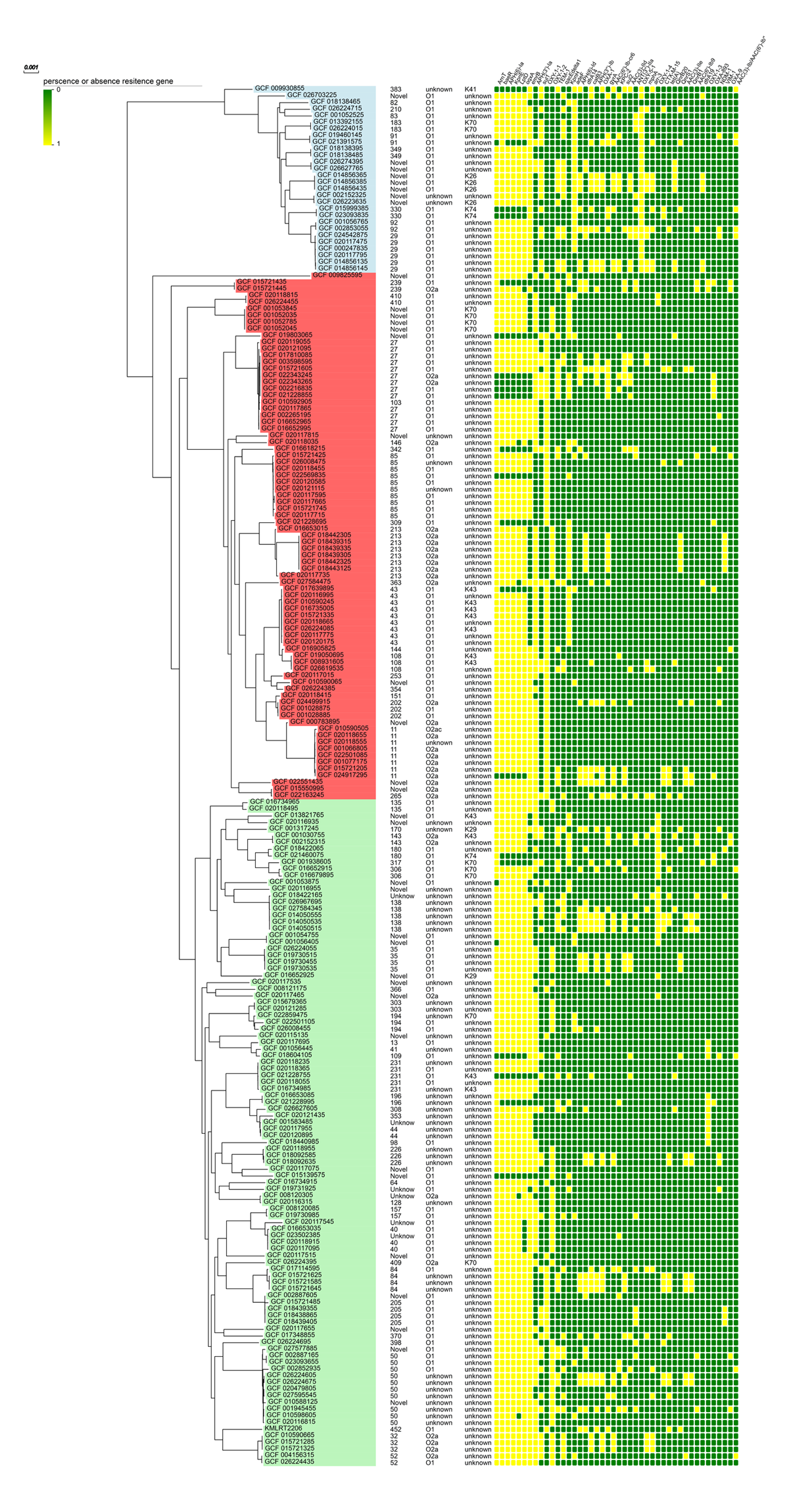

Supplement: Supplementary Figure 2 — Maximum-likelihood phylogenetic tree of K. michiganensis with a heatmap of antibiotic resistance gene. [file Image_2.tif]

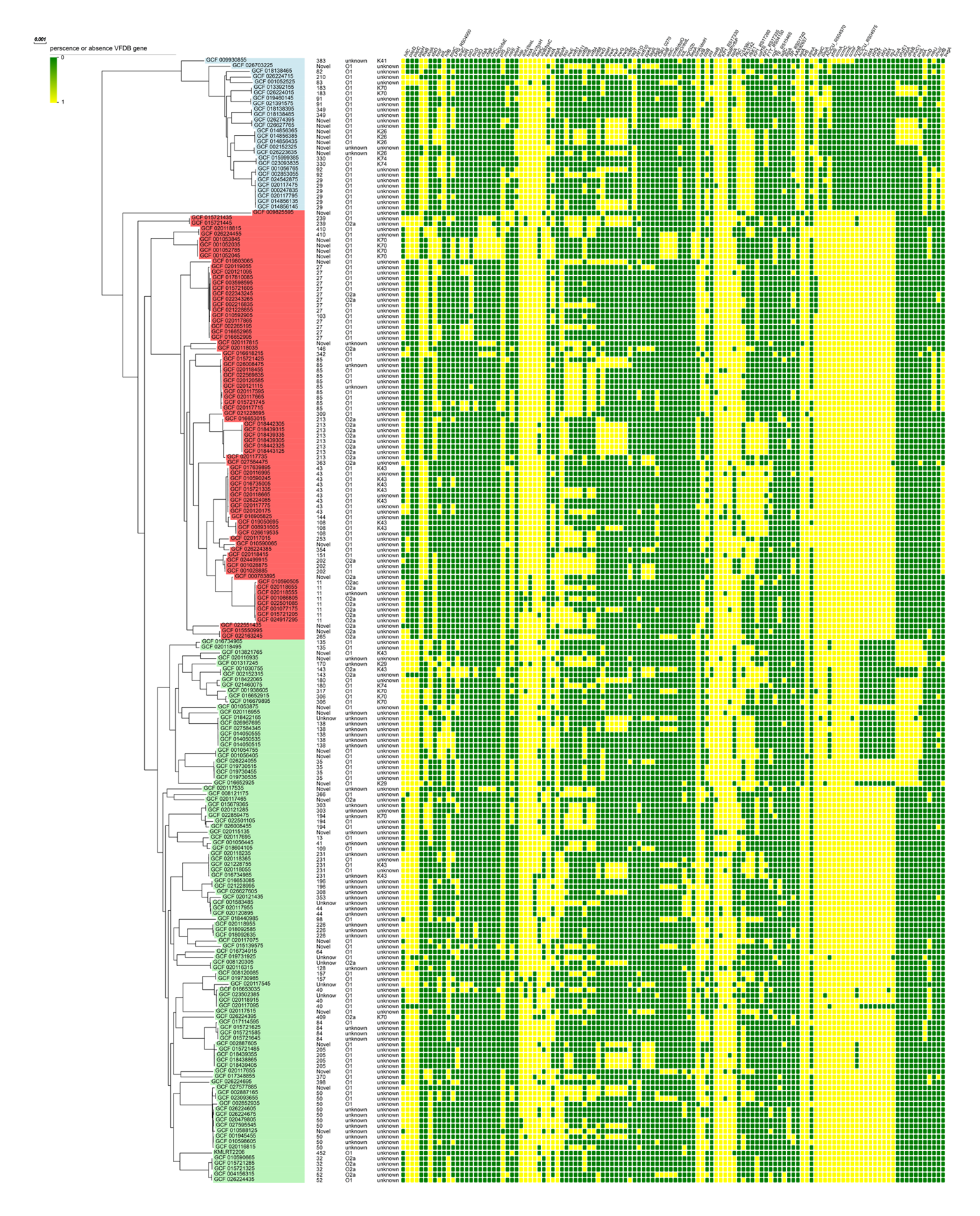

Supplement: Supplementary Figure 3 — Maximum-likelihood phylogenetic tree of K. michiganensis with a heatmap of virulence gene. [file Image_3.tif]
